# Association of Cancer—Associated Venous Thromboembolism with the Primary Site of Colorectal Cancer, with Respect to KRAS/NRAS/BRAF Mutations

**DOI:** 10.3390/biomedicines14020312

**Published:** 2026-01-30

**Authors:** Josipa Jović Zlatović, Milenko Bevanda, Vesna Telesmanić Dobrić, Zvonimir Curić, Inga Marijanović, Marija Karaga, Marko Skelin, Snježana Tomić, Ivo Dilber, Tomislav Omrčen

**Affiliations:** 1 Department of Hematology, Oncology, Allergology and Clinical Immunology, General Hospital of Sibenik-Knin County, 22000 Šibenik, Croatia; 2 School of Medicine, University of Mostar, 88000 Mostar, Bosnia and Herzegovina; 3 Professional Undergraduate Study Programme in Nursing, University of Applied Sciences Šibenik, 22000 Šibenik, Croatia; 4 Department of Gastroenterology, University Hospital Mostar, 88000 Mostar, Bosnia and Herzegovina; 5 Department of Oncology and Nuclear Medicine, General Hospital Zadar, 23000 Zadar, Croatia; 6 Department of Oncology, General Hospital Dubrovnik, 21000 Dubrovnik, Croatia; 7 Clinic of Oncology, University Clinical Hospital Mostar, 88000 Mostar, Bosnia and Herzegovina; 8 Pharmacy Department, General Hospital of Sibenik-Knin County, 22000 Šibenik, Croatia; 9 Department of Basic and Clinical Pharmacology with Toxicology, Faculty of Medicine, University of Rijeka, 51000 Rijeka, Croatia; 10 Department of Pathology, Citology and Forensic Medicine, University Hospital Center Split, 21000 Split, Croatia; 11 School of Medicine, University of Split, 21000 Split, Croatia; tomislavomrcen@yahoo.com; 12 Department of Oncology and Radiotherapy, University Hospital Split, 21000 Split, Croatia

**Keywords:** metastatic colorectal cancer, venous thromboembolism, tumor sidedness, KRAS, BRAF, Khorana score

## Abstract

**Background/Objectives:** Venous thromboembolism (VTE) is a common and clinically significant complication in patients with metastatic colorectal cancer (mCRC). Tumor sidedness and molecular alterations such as RAS and BRAF mutations are established prognostic factors in mCRC; however, their role in VTE risk stratification remains unclear. This study aimed to evaluate the association between primary tumor sidedness, KRAS/NRAS/BRAF mutational status, and VTE occurrence in patients with mCRC treated in the outpatient setting. **Methods**: This multicenter ambispective observational study included 224 patients with mCRC treated with first-line chemotherapy with or without targeted therapy. All patients had known KRAS/NRAS/BRAF statuses. The primary endpoint was the association between tumor sidedness and VTE risk. Secondary endpoints included associations between oncogenic mutations and VTE, subgroup analyses according to tumor localization and mutational status, and overall survival (OS). Multivariate logistic regression was used to identify independent predictors of VTE. **Results**: After a median follow-up of 21 months, VTE occurred in 23.3% of patients. The incidence of VTE was significantly higher in right-sided colorectal cancer (RCRC) compared with left-sided colorectal cancer (LCRC) (41.0% vs. 17.6%, *p* < 0.001). Although KRAS/NRAS and BRAF mutations were more frequent in RCRC, mutational status was not independently associated with VTE. In multivariate analysis, right-sided tumor location remained a strong predictor of VTE (OR 5.2; 95% CI 1.9–14.1; *p* = 0.001), along with anti-EGFR therapy. The Khorana score classified most patients as low risk and did not reliably identify those who developed VTE. VTE occurrence was not significantly associated with OS, whereas right-sided tumor location was associated with inferior survival. **Conclusions**: Right-sided tumor location is an independent predictor of VTE in patients with mCRC and confers a high absolute thrombotic risk not captured by the Khorana score. Incorporating tumor sidedness into VTE risk assessment may improve identification of patients who could benefit from primary thromboprophylaxis.

## 1. Introduction

Colorectal cancer (CRC) is the third most common malignancy and the second leading cause of cancer-related death worldwide. In 2022. more than 1.9 million new cases of CRC were diagnosed worldwide, with approximately 900,000 CRC-related deaths [1]. CRC accounts for 9.6% of all new cancer cases globally and 9.3% of all cancer deaths. In Europe, there were approximately 500,000 new cases and 243,000 deaths from CRC in 2018 [2]. About 20% of patients have metastatic disease at diagnosis, and approximately one-third of patients who present with localized disease will develop metastases, with 5-year survival rate ranging from 91% for localized disease to 14% for metastatic disease [3].

Over the last decade, colon tumor sidedness has been a topic of great interest. The literature suggests that right-sided colorectal cancer (RCRC) and left-sided colorectal cancer (LCRC)—defined by the primary location of CRC—represent two distinct diseases, differing in embryology, epidemiology, pathology, molecular pathways, clinical characteristics, treatment approaches, and overall survival [4,5,6,7]. RCRCs are more prevalent in females and older patients; they are more often diagnosed at an advanced or metastatic stage, with high-grade, mucinous histology, and are more commonly MSI-H (microsatellite instability-high), RAS-mutated (RASmt), or BRAF-mutated (BRAFmt) compared to LCRCs. Many studies have shown that patients with metastatic RCRC (cecum to transverse colon, RmCRC) have worse responses to treatment and lower overall survival (OS) than those with metastatic LCRC (descending colon to rectum, LmCRC) [7,8,9,10,11].

A recent retrospective study observed an association between right-sided CRC and an increased risk of venous thromboembolism (VTE), with a negative impact on survival [12]. However, the role of RAS/BRAF mutations and tumor sidedness in the risk of VTE in mCRC remains unclear. There are conflicting data regarding the role of KRAS mutations in the risk of VTE in patients with CRC, while NRAS and BRAF biomarkers have not been confirmed to be associated with an increased risk of VTE [12,13,14].

The primary aim of our study was to examine the impact of tumor sidedness and RAS/BRAF status on VTE occurrence in a cohort of patients with mCRC receiving outpatient anticancer therapy, to potentially inform the development of primary thromboprophylaxis protocols.

## 2. Materials and Methods

This was a multicenter, ambispective, observation study conducted at three oncologic centers in Croatia. Outpatients (N = 194) with mCRC and known KRAS/NRAS/BRAF oncogenic status, whose disease was diagnosed and treated with first-line chemotherapy with or without targeted therapy according to the physician’s decision, were retrospectively included in the study. We analyzed patients treated from June 2013 to April 2018. The prospective arm included patients (N = 40) who had signed informed consent. The prospective part of the trial was performed from April 2018 to March 2020, with a minimum follow-up of 1 year. Since the prospective arm of the trial was performed before the COVID-19 pandemic, we excluded the potential impact of COVID-19 pandemic on the incidence of VTE.

Exclusion and inclusion criteria: Eligible patients were aged ≥ 18 years with newly diagnosed advanced/metastatic or recurrent CRC, pathologically confirmed adenocarcinoma, and known KRAS/NRAS/BRAF mutational status. Patients who underwent surgical treatment of the primary tumor or metastases, or adjuvant/neoadjuvant chemotherapy and/or radiotherapy (RT) for localized disease, were eligible. Patients treated with induction first-line chemotherapy with or without epidermal growth factor receptor (EGFR) inhibitors (cetuximab or panitumumab) or vascular endothelial growth factor (VEGF) inhibitor (bevacizumab) were included in this analysis. The minimum treatment duration was 6 months. Exclusion criteria were: lack of KRAS/NRAS/BRAF mutational status; lack of pre-chemotherapy laboratory results and body mass index (BMI) severe renal impairment [creatinine clearance (CrCl) < 30 mL/min]; previous VTE superficial thrombophlebitis or arterial thrombosis; anticoagulant therapy for other indications.

Data collection. Demographic data (gender, age), basic clinical characteristics (pre-chemotherapy blood count, comorbidities, tumor localization, smoking history, performance status, BMI history of previous VTE, type of antineoplastic therapy) and pathological characteristics (tumor grade, nodal status, oncogenic mutations) were collected at the time of mCRC diagnosis through medical history review. The mutational status of RAS and BRAF was determined using the polymerase chain reaction (PCR) method with internationally certified tests: cobas^®^ DNA Sample Preparation Kit (Roche, Basel, Switzerland) for DNA isolation from paraffin-embedded tissue, KRAS Mutation Test v2 (Roche), and BRAF/NRAS Mutation Test (Roche). Tumor staging was assigned according to the Tumor, Node and Metastasis (TNM) Classification of Malignant Tumors, eighth edition [15]. VTE risk factors considered were gender, BMI, age at diagnosis, performance status, arterial hypertension, diabetes mellitus, smoking history, presence of central venous catheters, venous varices, tumor grade, site of metastases, oncogenic mutations, type of chemotherapy, VEGF inhibitors, EGFR inhibitors, transfusions, megestrol acetate, and Khorana risk score. The Khorana risk score was calculated for all patients using pre-chemotherapy laboratory results and BMI. We have calculated, based on the CASSINI and AVERT trials with a cutoff value of 2 (low risk of VTE < 2 and high risk of VTE ≥ 2) [16,17].

VTE was defined as DVT involving the upper or lower extremity, pelvis, or catheter-related thrombosis, and/or symptomatic or asymptomatic pulmonary embolism (PE). Time to VTE (TTVTE) was measured from 6 months before diagnosis until the first venous event after the diagnosis of mCRC. Doppler ultrasonography and CT pulmonary angiography were used to confirm suspected DVT or PE. VTE was defined as deep vein thrombosis (DVT) involving the upper or lower extremity, pelvis, or catheter-related thrombosis, and/or symptomatic or asymptomatic pulmonary embolism (PE). Time to VTE (TTVTE) was measured from 6 months before diagnosis until the first venous event after the diagnosis of mCRC. Doppler ultrasonography and CT pulmonary angiography were used to confirm suspected DVT or PE [18].

Tumor sidedness: The anatomic site of CRC was categorized by primary location according to the ICD-O-3 codes (International Classification of Diseases for Oncology, third edition) [19]. Primary tumors located in the rectum, rectosigmoid, sigmoid colon, descending colon, and splenic flexure were defined as LCRC, whereas primary tumors located in the transverse colon, hepatic flexure, ascending colon, and cecum were defined as RCRC.

Clinical outcomes:

The primary endpoint was the association between the primary tumor location of CRC and VTE risk.

The secondary endpoints were:

1. The association between KRAS/NRAS/BRAF mutational status and VTE risk.
2. Additional subgroup analyses of the VTE rate according to tumor localization in cohorts with different KRAS/NRAS/BRAF status in patients with mCRC.
3. Overall survival (OS), calculated from the start of treatment for mCRC until death or last follow-up.

The primary endpoint was the association between the primary tumor location of CRC and VTE risk.

The secondary endpoints were:

1. The association between KRAS/NRAS/BRAF mutational status and VTE risk.
2. Additional subgroup analysis of the VTE rate according to tumor localization in cohorts with different KRAS/NRAS/BRAF status.
3. Overall survival (OS), calculated from diagnosis of mCRC until death or last follow-up.

Statistical analysis:

Categorical variables were presented as absolute and relative frequencies and compared using the Chi-square or Fisher’s exact test, as appropriate. The distribution of continuous variables was assessed with the Shapiro–Wilk test, and they were summarized using median and interquartile range. Differences between two independent groups were analysed using the Mann–Whitney U test. Independent factors associated with VTE were identified using logistic regression (bivariate and multivariate, stepwise method). Kaplan–Meier analysis with a log-rank test was conducted to compare survival between groups. All *p*-values were two-tailed, and statistical significance was defined as α = 0.05. Statistical analyses were performed using MedCalc^®^ Statistical Software, version 23.1.7 (MedCalc^®^ Software Ltd., Ostend, Belgium; https://www.medcalc.org; 2025).

This study was conducted in accordance with the Declaration of Helsinki and approved by the Ethics Committees of the General Hospital of Sibenik-Knin County (No. 01-2570/1-18),General Hospital Zadar (No. 01-1971/18-3/18), General Hospital Dubrovnik (No 01-49/4.16-20).

## 3. Results

### 3.1. The Association Between the Primary Tumor Location of CRC and VTE Risk

Of 234 patients, a total of 224 (N = 184 in retrospective and N = 40 in prospective arm) were eligible for this study. Of 224 patients, 24.1% (N = 54) had RCRC and 75.9% (N = 170) had LCRC. The median age was 67 years (IQR 61–74); 95.1% (N = 213) had a good performance status (ECOG PS 0-1), and 67.3% (N = 150) were male. All patients (100%, N = 224) were treated with chemotherapy; 80.2% (N = 133) received bevacizumab-containing regimens, and 19.8% (N = 33) received anti-EGFR therapy. Patients with RCRC were more commonly treated with bevacizumab (91.9% vs. 76.9%, *p* = 0.04), while those with LCRC more frequently received anti-EGFR therapy (23.1% vs. 8.1%). Patients with RCRC were older (Mann–Whitney U test, *p* = 0.008). KRAS/NRASmt were detected in 50% (N = 112), NRASmt in 2.7% (N = 6), BRAFmt in 4.5% (N = 10), and KRAS/NRAS/BRAF wild-type status (wt) in 42.9% (N = 96) of patients. KRAS/NRASmt were more common in RCRC than in LCRC (61.1% vs. 50% *p* = 0.003), as were BRAFmt (11.1% vs. 2.4% *p* =0.003). Relevant baseline patient characteristics between the two groups are summarized in Table 1.

The median follow-up time was 21 months (IQR 13–33) with a total of 23.3% VTE events (N = 43 in retrospective arm and N = 9 in prospective arm). The incidence of VTE was 41% (N = 22) in RCRC patients and 17.6% (N = 32) in LCRC patients (*p* < 0.001). According to mutational status, the incidence of VTE was 46% (N = 24) in KRAS/NRASmt patients, 9.6% (N = 5) in BRAFmt, and 44% (N = 23) in KRAS/NRAS/BRAFwt patients. Patients with VTE and RCRC had significantly more KRAS/NRASmt (57.7% vs. 40%, *p* = 0.001) and BRAFmt (23.1% vs. 0%, *p* = 0.001) than patients with VTE and LCRC (Table 2).

The highest incidence of thromboembolism during the first year of follow-up is shown in Table 3.

A multivariate logistic regression analysis using a backward stepwise method was performed to identify independent predictors of VTE. Variables included in the model were transfusion requirement, Granulocyte-Colony Stimulating Factor (G-CSF) use, corticosteroids, megestrol acetate, type of chemotherapy, type of biological therapy, tumor localization, and oncogenic status. The model was additionally adjusted for smoking, diabetes mellitus, arterial hypertension, venous varices, antiplatelet therapy, and comorbidity. In the multivariate analysis, only right-sided tumor localization (OR = 5.01; 95% CI: 1.94 to 12.93) and the use of EGFR targeted therapy compared with bevacizumab (OR = 4.27; 95% CI: 1.62 to 11.3) remained independently associated with VTE. The overall model was statistically significant (Chi-squared test = 29.2; *p*-value = 0.001) and explained 16% to 25% of the variance in VTE occurrence (Cox & Snell R^2^ to Nagelkerke R^2^) (Table 4).

### 3.2. The Rate of VTE According to Tumor Localization in Cohorts with Different KRAS/NRAS/BRAF Status

The median follow-up time was 21 months (IQR 13–33). Of the total 22.7% (N = 29) of patients with VTE and KRAS/NRAS/BRAFmt, there were significantly more from the group with right-sided tumors (43.6%, N = 17; chi-squared test, *p* < 0.001; see Table 5). Multivariate stepwise logistic regression analysis, adjusted for independent baseline predictors of VTE (including venous varices, comorbidities, targeted therapy, type of targeted therapy, chemotherapy, oncogene mutations and tumor sidedness), confirmed that right-sided tumors were significant predictors of VTE in KRAS/NRAS/BRAFmt patients (OR = 5.75; 95% CI: 1.68–19.72 *p* = 0.005) compared to the left-sided tumors (Table 6).

### 3.3. Overall Survival (OS) and VTE

**Figure 1:**
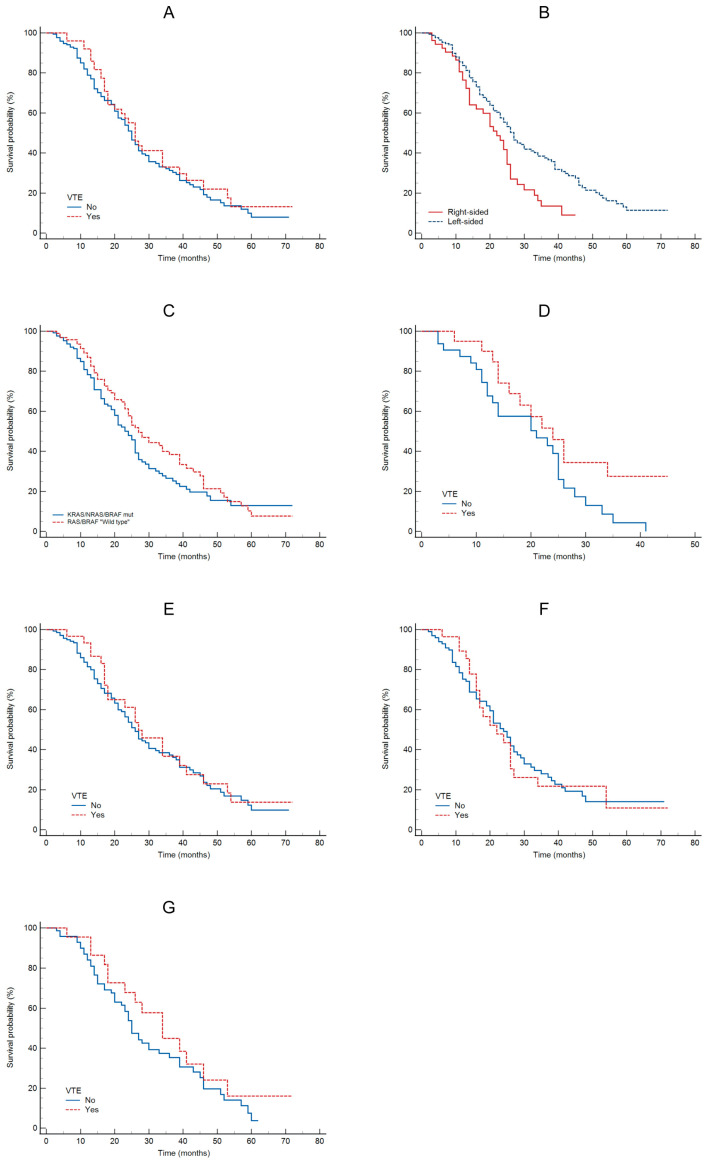
OS according: to VTE (**A**); to tumor sidedness (**B**); to oncogene status (**C**); to VTE in patients with RCRC (**D**); to VTE in patients with LCRC (**E**); to VTE in patients with KRAS/NRASmt (**F**); to VTE in patients with KRAS/NRAS/BRAFwt (**G**).

The Kaplan–Meier analysis showed no significant association between VTE and OS (HR = 1.17; 95% CI: 0.8–1.7; *p* = 0.4; see Figure 1A). This analysis indicated a significant association between tumor sidedness and OS, with 27 months for left-sided tumors and 22 months for right-sided tumors (HR = 1.85; 95% CI: 1.2–2.8; *p* = 0.005; see Figure 1B). Compared to KRAS/NRAS/BRAFmt vs. wild type patients, there was no significant difference in OS (HR = 1.27; 95% CI: 0.9–1.7; *p* = 0.15, see Figure 1C), although wild type patients had numerically longer OS. No statistically significant difference in OS was observed between patients with or without VTE for right-sided tumors (HR = 1.89; 95% CI: 0.9–3.6; *p* = 0.05; see Figure 1D) or left-sided tumors (HR = 1.13; 95% CI: 0.7–1.8; *p* = 0.6; see Figure 1E). OS was 24 months for left-sided and 22 months for right-sided KRAS/NRASmt tumors (HR = 0.99; 95% CI: 0.84–2.16; *p* = 0.96; see Figure 1F). No statistically significant difference in OS was observed between patients with or without VTE for KRAS/RAS/BRAFwt tumors (HR = 1.40; 95% CI: 0.8–2.4; *p* = 0.21; see Figure 1G).

## 4. Discussion

VTE is a frequent and clinically relevant complication in patients with mCRC [20,21,22,23].

In our multicenter ambispective study, nearly one quarter of patients (23.3%) developed VTE, confirming the substantial thrombotic burden in this population. Importantly, our data demonstrate that RCRC is associated with a significantly higher absolute risk of VTE compared with left-sided disease, independently of established clinical risk factors and oncogenic mutations. We observed a markedly higher cumulative incidence of VTE in patients with RCRC (41%) compared with those with LCRC (17.6%). This difference corresponds to a clinically meaningful absolute risk increase of more than 20%, which remained significant after multivariate adjustment. Right-sided tumor localization emerged as one of the strongest independent predictors of VTE (OR 5.2), exceeding the predictive strength of most traditional clinical variables. These findings are consistent with emerging population-based and registry data suggesting that RCRC carries a higher thrombotic burden [11]. The biological plausibility is supported by known differences between right- and left-sided tumors, including higher rates of advanced stage, mucinous histology, peritoneal involvement, and adverse molecular profiles, all of which may contribute to a prothrombotic phenotype [9,13,24,25,26,27]. From a clinical perspective, tumor sidedness could represent a readily available, non-invasive variable that may help refine thrombotic risk assessment in routine practice.

The Khorana score remains the most widely used tool for predicting cancer-associated thrombosis in ambulatory patients receiving chemotherapy [21,22]. However, in our cohort, the majority of patients were classified as low risk according to the Khorana score, with more than 96% of patients having a score < 2. Despite this, a substantial proportion of these “low risk” patients developed VTE, highlighting a discordance between predicted and observed absolute risk. Notably, the Khorana score did not significantly discriminate between patients with and without VTE in our study, nor did it capture the pronounced difference in VTE incidence between right- and left-sided tumors. This limitation likely reflects the design of the Khorana score, which assigns colorectal cancer to a low-risk category and relies heavily on baseline laboratory parameters and BMI, without incorporating tumor biology or anatomic characteristics. Our findings therefore support growing evidence that the Khorana score may underestimate VTE risk in selected subgroups of mCRC patients, particularly those with right-sided tumors. This has important clinical implications, as reliance on the Khorana score alone may result in missed opportunities for primary thromboprophylaxis in patients with a high absolute risk of thrombosis.

Although univariate analyses suggested higher VTE rates among patients with KRAS/NRAS or BRAF mutations, oncogenic status did not remain independently associated with VTE after multivariate adjustment. This suggests that tumor sidedness may act as a stronger surrogate marker of thrombotic risk than individual molecular alterations in this setting. Nevertheless, the numerically higher VTE incidence observed in BRAF-mutated tumors warrants further investigation, particularly given emerging data linking BRAF mutations, tissue factor expression, and aggressive tumor biology [13,24,25,26,27,28].

Our study also identified anti-EGFR therapy as an independent predictor of VTE, particularly in patients with left-sided tumors. This observation is clinically relevant, as EGFR inhibitors are preferentially used in left-sided, RAS wild-type disease according to current treatment guidelines. The interaction between tumor sidedness, treatment selection, and thrombosis risk further underscores the complexity of VTE pathogenesis in mCRC and the need for individualized risk assessment.

Taken together, our results suggest that absolute VTE risk in mCRC is driven not only by traditional clinical factors but also by tumor-specific characteristics, particularly primary tumor location. The high absolute VTE risk observed in patients with RCRC—despite low Khorana scores—raises the question of whether this subgroup may benefit from tailored thromboprophylaxis strategies. Current international guidelines do not recommend routine primary thromboprophylaxis for most ambulatory patients with CRC, largely because of the perceived low baseline risk. However, our findings indicate that patients with RCRC represent a distinct high-risk subgroup that is not adequately captured by existing risk models. Incorporating tumor sidedness into future risk stratification tools may improve patient selection for prophylactic anticoagulation and help balance thrombotic and bleeding risks more effectively.

The limitations of our study include its partially retrospective design, which may introduce selection bias and limit causal conclusions. The relatively small number of RCRC patients (24%) may reduce the generalizability and precision of the findings related to RCRC. Additionally, we were unable to account for all potential risk factors for VTE, such as laboratory markers and concomitant antithrombotic therapies, which were not systematically recorded in the retrospective cohort. The study’s focus on three Croatian oncology centers limits the external validity, and the lack of long-term follow-up data on survival and VTE recurrence prevents a more comprehensive understanding of the long-term impact of VTE in this population.

Future prospective studies with larger and more diverse cohorts are needed to validate tumor sidedness as a predictor of VTE and to assess its integration with established models such as the Khorana score. Importantly, randomized trials are warranted to evaluate whether patients with right-sided mCRC and high absolute VTE risk benefit from primary thromboprophylaxis, even when classified as low risk by conventional scores.

## 5. Conclusions

In conclusion, our study identifies primary tumor sidedness as a key determinant of venous thromboembolism risk in patients with metastatic colorectal cancer. Right-sided tumors were associated with a markedly increased absolute risk of VTE, independent of established clinical risk factors, oncogenic mutations, and current risk stratification models. Importantly, this excess risk was not adequately captured by the Khorana score, underscoring its limited discriminatory ability in this specific population. These findings suggest that tumor sidedness reflects underlying biological and treatment-related factors that substantially influence thrombotic risk. Incorporation of tumor location into future VTE risk assessment models may improve identification of high-risk patients and enable more personalized thromboprophylaxis strategies. Ultimately, prospective studies and randomized trials are warranted to determine whether patients with right-sided metastatic colorectal cancer derive clinical benefit from tailored primary thromboprophylaxis.

## Figures and Tables

**Table 1:** Baseline characteristics of the studied population according to primary tumor localization.

	Right Sided (*n* = 54)	Left Sided (*n* = 170)	Total (*n* = 224)	p *
Gender (Male) [*n* (%)]	33 (61.1)	117 (68.8)	150 (67)	0.29
Age [median (IQR)]	71 (64–75)	66 (59–72)	67 (60–74)	**0.005** ^†^
BMI [median (IQR)]	24.6 (23.0–27.8)	25.3 (22.9–28.7)	25.0 (23–28.2)	0.59 ^†^
BMI (≥30 kg/m^2^) [*n* (%)]	6 (11.1)	32 (18.8)	38 (17)	**0.04**
ECOG PS at the time of diagnosis, [*n* (%)]				
0	19 (35.2)	96 (56.5)	115 (51.3)	**0.02**
1	31 (57.4)	67 (39.4)	98 (43.8)	
2	4 (7.4)	7 (4.1)	11 (4.9)	
History of smoking [*n* (%)]	10 (18.5)	52 (30.6)	62 (27.7)	0.08
Diabetes mellitus [*n* (%)]	8 (14.8)	38 (22.4)	46 (20.5)	0.23
Arterial hypertension [*n* (%)]	25 (46.3)	82 (48.2)	107 (47.8)	0.80
Varicose vein [*n* (%)]	10 (18.5)	26 (15.3)	36 (16.1)	0.57
Antiplatelet therapy [*n* (%)]	12 (22.2)	27 (15.9)	39 (17.4)	0.28
Follow-up (months) [median (IQR)]	20 (11–26)	23 (14–36)	21 (13–33)	**0.03** ^†^
Outcome [*n* (%)]				
Lives	13 (24.1)	55 (32.4)	68 (30.4)	0.25
Death	41 (75.9)	115 (67.6)	156 (69.6)	
Grade [*n* (%)]				
low	4 (8.3)	17 (10.9)	21 (10.3)	0.60
Intermediate	29 (60.4)	101 (64.7)	130 (63.7)	
High	15 (31.3)	38 (24.4)	53 (26)	
Khorana score (*n* = 216) [*n* (%)]				
0	19 (35.8)	104 (63.8)	123 (56.9)	**0.001**
1–2	32 (60.4)	54 (33.1)	86 (39.8)	
3≥	2 (3.8)	5 (3.1)	7 (3.9)	
Khorana score (*n* = 216) [*n* (%)]				
0–1	51 (96.2)	158 (96.9)	209 (96.8)	0.80
2≥	2 (3.8)	5 (3.1)	7 (3.2)	
KRAS/NRAS/BRAF [*n* (%)]				
KRAS/NRASmt	33 (61.1)	85 (50)	118 (52.7)	**0.003**
BRAFmt	6 (11.1)	4 (2.4)	10 (4.5)	
RAS/BRAFwt	15 (27.8)	81 (47.6)	96 (42.9)	
Mucinous histology [*n* (%)]	9 (17)	17 (10.1)	26 (11.8)	0.18
Chemotherapy [*n* (%)]				
Folfiri/Xeliri	26 (49.1)	90 (53.3)	116 (52.3)	0.70
Folfox/Xelox	17 (32.1)	55 (32.5)	72 (32.5)	
Capecitabin	10 (18.9)	24 (14.2)	34 (15.3)	
Type of targeted therapy [*n* (%)]				
Bevacizumab	34 (91.9)	100 (76.9)	134 (80.2)	**0.04**
EGFR inhibitor (cetuximab/panitumumab)	3 (8.1)	30 (23.1)	33 (19.8)	
Surgery of the primary tumor [*n* (%)]	47 (87)	151 (88.8)	198 (88.4)	0.72
Metastatic localization [*n* (%)]				
Liver	23 (42.6)	76 (44.7)	99 (44.2)	0.25
Lungs	4 (7)	22 (13)	26 (11.6)	
Peritoneum	10 (18.5)	16 (9.4)	26 (11.6)	
Other	17 (31.5)	56 (32.9)	73 (32.6)	
Metastasectomy	9 (16.7)	52 (30.6)	61 (27.2)	**0.04**

* Chi-squared test; ^†^ Fisher’s Exact Test Chi-squared test; ^†^ as appropriate. Data presented as the median (interquartile range) or n (%). Abbreviations: BMI: body mass index, Abbreviations: BRAF: v-Raf Murine Sarcoma Viral Oncogene Homolog B1, RASB-Raf proto-oncogene serine/threonine kinas mutated, RAS: Rat Sarcoma Virus, ECOG PS: Eastern Cooperative Oncology Group; EGFR: Epidermal Growth Factor Receptor. Values in bold indicate statistically significant differences (*p* < 0.05).

**Table 2:** Patient’s characteristics according to VTE.

	VTE, No (*n* = 171)	VTE, Yes (*n* = 52)	Total (*n* = 223)	p *
Gender, (M) [*n* (%)]	117 (68.4)	33 (63.5)	150 (67.3)	0.51
Age [median (IQR)]	68 (61–74)	66 (60–71)	67 (60–74)	0.11 ^‡^
BMI (kg/m^2^) [median (IQR)]	25.1 (23.0–27.9)	24.7 (22.2–28.9)	25.0 (23–28.2)	0.56 ^‡^
BMI (>30 kg/m^2^) [*n* (%)]	30 (17.5)	8 (15.4)	38 (17)	0.88
ECOG PS at the time of diagnosis, [*n* (%)]				
0–1	164 (95.9)	48 (92.3)	212 (95.0)	0.38
2	7 (4.1)	4 (7.7)	11 (4.9)	
Khorana score [*n* (%)]				
0	97 (58.8)	26 (51)	123 (56.9)	0.44
1–2	62 (37.6)	24 (47.1)	86 (39.8)	
3≥	6 (3.6)	1 (2)	7 (3.2)	
Khorana score [*n* (%)]				
0–2	159 (96.4)	50 (98)	209 (96.8)	>0.99
3≥	6 (3.6)	1 (2)	7 (3.2)	
History of smoking, [*n* (%)]	54 (31.6)	8 (15.4)	62 (27.8)	**0.02**
Diabetes mellitus (DM) [*n* (%)]	36 (21.1)	10 (19.2)	46 (20.6)	0.78
Arterial hypertension [*n* (%)]	85 (49.7)	22 (42.3)	107 (48)	0.35
Varicose vein [*n* (%)]	31 (18.1)	5 (9.6)	36 (16.1)	0.14
Antiplatelet therapy, [*n* (%)]	31 (18.1)	8 (15.4)	39 (17.5)	0.65
Localization [*n* (%)]				
Right sided	32 (18.7)	22 (42.3)	54 (24.2)	**0.001**
Left sided	139 (81.3)	30 (57.7)	169 (75.8)	
Oncogene status [*n* (%)]				
KRAS/NRASmt	94 (94.9)	24 (82.7)	118 (92.2)	**0.04**
BRAFmt	5 (5.1)	5 (17.3)	10 (7.8)	
Chemotherapy [*n* (%)]				
Folfiri/Xeliri	84 (50)	31 (59)	115 (52)	0.45
Folfox/Xelox	58 (34)	14 (26)	72 (32)	
Capecitabin	27 (16)	7 (15)	34 (16)	
Targeted therapy [*n* (%)]	129 (76.3)	36 (69.2)	165 (74.7)	0.30
Targeted therapy [*n* (%)]				
Bevacizumab	109 (84.5)	24 (64.9)	133 (80.1)	**0.008** ^†^
EGFR inhibitor (cetuximab/panitumumab)	20 (15.5)	13 (35.1)	33 (19.9)	
Mucinous histology [*n* (%)]				
Yes	148 (88.1)	46 (88.5)	194 (88.2)	0.94
No	20 (11.9)	6 (11.5)	26 (11.8)	
Transfusion [*n* (%)]	36 (21)	11 (21)	47 (21)	0.99
G-CFS [*n* (%)]	45 (26)	14 (27)	59 (27)	0.97
Megestrol acetate [*n* (%)]	18 (11)	2 (4)	20 (9)	0.17 ^†^
Central venous catheter (CVI) [*n* (%)]	19 (11)	8 (15)	27 (12)	0.42

* Chi-squared test; ^†^ Fisher’s Exact Test. ^‡^ Mann–Whitney U test. Data presented as the median (interquartile range) or n (%). Abbreviations: BRAF: v-Raf Murine Sarcoma Viral Oncogene Homolog B1, RASB-Raf proto-oncogene serine/threonine kinas mutated, RAS: Rat Sarcoma Virus, ECOG PS: Eastern Cooperative Oncology Group; EGFR: Epidermal Growth Factor Receptor, G-CFS: granulocyte-colony called a growth factor. The Total column is provided for descriptive purposes only. *p*-values refer to comparisons between VTE and non-VTE groups. Values in bold indicate statistically significant differences (*p* < 0.05).

**Table 3:** VTE characteristics according to tumor sidedness.

	Number (%)			p *
	Right Sided (*n* = 22)	Left Sided (*n* = 30)	Total (*n* = 52)	
Time to VTE (TTVTE)				
6 months before diagnosis	2 (9.1)	1 (3.3)	3 (5.8)	0.62
6 months after diagnosis	9 (40.9)	10 (33.3)	19 (36.6)	
6–12 months after diagnosis	11 (50)	19 (63.4)	30 (57.7)	
Localization				
VTE without PE	13 (87)	18 (95.0)	31 (91)	0.57
VTE with PE	2 (13)	1 (5.0)	3 (9)	

* Fisher’s Exact Test; Abbreviations: TTVTE: Time to venous thromboembolism, PE embolio pulmonum. The Total column is provided for descriptive purposes only. *p*-values refer to comparisons between VTE and non-VTE groups.

**Table 4:** Predictors of venous thromboembolism (VTE): bivariate and multivariate logistic regression analysis.

	Bivariate Logistic Regression		* Multivariate Logistic Regression (Stepwise)	
	*p*-Value	OR (95% CI)	*p*-Value	OR (95% CI)
Transfusions	0.98	0.99 (0.47–2.14)	-	-
G-CSF	0.97	1.02 (0.50–5.05)	-	-
Corticosteroids	0.75	1.32 (0.25–7.01)	-	-
Megestrol acetate	0.16	0.34 (0.08–1.51)	-	-
Chemotherapy (Folfiri/Xeliri)				
Folfox/Xelox	0.24	0.65 (0.32–1.34)	-	-
Capecitabin	0.46	0.70 (0.28–1.78)	-	-
Targeted therapy (EGFR inhibitor vs. Bevacizumab)	**0.01**	2.95 (1.29–6.75)	**0.003**	4.27 (1.62–11.3)
Localization (right sided vs. left sided)	**0.001**	3.19 (1.63–6.23)	**0.001**	5.01 (1.94–12.93)
KRAS/NRAS/BRAFmt	0.79	1.09 (0.58–2.04)	-	-

* Adjusted for smoking, diabetes mellitus, arterial hypertension, venous varices, antiplatelet therapy and comorbidity. Abbreviation: VTE—venous thromboembolism; OR—Odds Ratio; CI—Confidence Interval. Values in bold indicate statistically significant differences (*p* < 0.05).

**Table 5:** Association of tumor sidedness and RAS/BRAF status with VTE * (Logistic regression analysis).

	Number (%) of Patients According to Tumor			p *
	Right	Left	Total	
VTE—No	22 (56.4)	77 (86.5)	99 (77.3)	**<0.001**
VTE—Yes	17 (43.6)	12 (13.5)	29 (22.7)	
Total	39 (100)	89 (100)	128 (100)	

* Chi-squared Test. Values in bold indicate statistically significant differences (*p* < 0.05).

**Table 6:** Overall survival and VTE.

	Median	95% CI	Log-Rank Test	Hazard Ratio (95% CI)
Overall survival (OS)	25	23–27	-	-
VTE				
No	25	21–27	0.40	1.17 (0.8 to 1.7)
Yes	26	18–34		
Tumor location				
Right	22	14–25	**0.005**	1.85 (1.2 to 2.8)
Left	27	23–30		
Oncogene status				
KRAS/NRAS/BRAFmt	24	20–26	0.15	1.27 (0.9–1.7)
KRAS/NRAS/BRAFwt	27	24–36		
Right				
VTE—no	21	12–25	0.05	1.89 (0.9–3.6)
VTE—yes	24	14–34		
Left				
VTE—no	26	22–30	0.60	1.13 (0.7–1.8)
VTE—yes	27	18–41		
KRAS/NRASmut				
VTE—no	24	20–27	0.96	0.99 (0.6–1.6)
VTE—yes	22	16–26		
KRAS/NRAS/BRAFwt				
VTE—no	25	22–33	0.21	1.40 (0.8–2.4)
VTE—yes	34	18–46		

Median values are expressed in months. Values in bold indicate statistically significant differences (*p* < 0.05).

## Data Availability

The data presented in this study are available on request from the corresponding author due to confidentiality agreements.
